# Divergent trends in ischaemic heart disease and stroke mortality in India from 2000 to 2015: a nationally representative mortality study

**DOI:** 10.1016/S2214-109X(18)30242-0

**Published:** 2018-08-01

**Authors:** Calvin Ke, Rajeev Gupta, Denis Xavier, Dorairaj Prabhakaran, Prashant Mathur, Yogeshwar V Kalkonde, Patrycja Kolpak, Wilson Suraweera, Prabhat Jha

**Affiliations:** Institute of Health Policy, Management and Evaluation University of Toronto, Toronto, ON, Canada; Department of Medicine, University of Toronto, Toronto, ON, Canada; Centre for Global Health Research, St Michael’s Hospital, and Dalla Lana School of Public Health, University of Toronto, Toronto, ON, Canada; Academic Research Development Unit, Rajasthan University of Health Sciences, Jaipur, Rajasthan, India; St John’s Medical College and Research Institute, Bangalore, India; Public Health Foundation of India, Gurugram, Haryana, India; National Centre for Diseases Informatics & Research, Indian Council of Medical Research, Bangalore, India; Society for Education, Action and Research in Community Health, Gadchiroli, Maharashtra, India; Centre for Global Health Research, St Michael’s Hospital, and Dalla Lana School of Public Health, University of Toronto, Toronto, ON, Canada

## Abstract

**Introduction:**

India accounts for about a fifth of cardiovascular deaths globally, but nationally representative data on mortality trends are not yet available. In this nationwide mortality study, we aimed to assess the trends in ischaemic heart disease and stroke mortality over 15 years using the Million Death Study.

**Methods:**

We determined national and subnational cardiovascular mortality rates and trends by sex and birth cohort using cause of death ascertained by verbal autopsy from 2001 to 2013 among 2·4 million households. We derived mortality rates for ischaemic heart disease and stroke by applying mortality proportions to UN mortality estimates for India and projected the rates from 2000 to 2015.

**Findings:**

Cardiovascular disease caused more than 2·1 million deaths in India in 2015 at all ages, or more than a quarter of all deaths. At ages 30–69 years, of 1·3 million cardiovascular deaths, 0·9 million (68·4%) were caused by ischaemic heart disease and 0·4 million (28·0%) by stroke. At these ages, the probability of dying from ischaemic heart disease increased during 2000–15, from 10·4% to 13·1% in men and 4·8% to 6·6% in women. Ischaemic heart disease mortality rates in rural areas increased rapidly and surpassed those in urban areas. By contrast, the probability of dying from stroke decreased from 5·7% to 5·0% in men and 5·0% to 3·9% in women. A third of premature stroke deaths occurred in the northeastern states, inhabited by a sixth of India’s population, where rates increased significantly and were three times higher than the national average. The increased mortality rates of ischaemic heart disease nationally and stroke in the northeastern states were higher in the cohorts of adults born in the 1970s onwards, than in earlier decades. A large and growing proportion of the ischaemic heart disease nationally and stroke deaths in high-burden states reported earlier diagnosis of cardiovascular disease, but low medication use.

**Interpretation:**

The unexpectedly diverse patterns of cardiovascular mortality require investigation to identify the role of established and new cardiovascular risk factors. Secondary prevention with effective and inexpensive long-term treatment and adult smoking cessation could prevent substantial numbers of premature deaths. Without progress against the control of cardiovascular disease in India, global goals to reduce non-communicable diseases by 2030 will be difficult to achieve.

**Funding:**

Fogarty International Center of the US National Institutes of Health, Dalla Lana School of Public Health, University of Toronto, Indian Council of Medical Research, and the Disease Control Priorities.

## Introduction

Cardiovascular disease, comprising mostly ischaemic heart disease and stroke, is the leading cause of death worldwide, accounting for 17·7 million deaths annually.^1^ Of these deaths, 6·2 million (35%) occur in middle age (30–69 years).^1^ WHO estimates that India accounts for just over a fifth of these deaths^1^ and therefore reduction of global cardiovascular mortality greatly depends on India, where cardiovascular disease develops a decade earlier in life compared with high-income countries.^2^

Cardiovascular mortality has not been measured directly and systematically across India, with evidence on burdens mostly drawn from small, local studies.^2–4^ Over the past 15 years, unequal distribution of the country’s rapid economic growth and urbanisation has probably contributed to marked regional differences in the key risk factors for cardiovascular mortality.^5^ Understanding of the differences in cardiovascular mortality by sex, rural and urban residence, and age across India can help to plan prevention and treatment services and identify causes that might differ from cardiovascular risk factors documented in high-income countries.^6^ We aimed to examine the trends in ischaemic heart disease and stroke mortality using the Million Death Study, a large ongoing nationally representative mortality study.^7^

## Methods

### Study design and setting

In India, most deaths occur at home and are not captured in official records. To generate reliable vital statistics, the Registrar General of India (RGI) established the Sample Registration System in 1971.^8^ After every decennial census, the RGI partitions India into 1 million small units, each of about 150–300 households. The RGI then selects a random sample of these units to monitor deaths over the next decade. 6671 and 7597 units were selected after the 1991 and 2001 censuses, respectively, representing the 2·4 million households included in the Million Death Study. This study collected information on deaths in 2001–03 and 2004–14, based on the 1991 and 2001 censuses, respectively. Every 6 months during the study period, 900 trained non-medical RGI surveyors investigated each death by interviewing a household member of the deceased using a modified WHO verbal autopsy questionnaire.^9^ The structured questionnaire captures awareness of any previous physician-diagnosed condition such as heart disease and stroke, and in 2004 we modified the questionnaire to include medication use. The verbal autopsy also includes a standard list of cardinal symptoms for the interviewer to elicit a chronological narrative of events preceding the death, written in the local language. Two trained physicians are randomly selected to review each questionnaire from those who read the relevant language, out of a pool of 400. Each independently determines the most probable underlying cause of death using an electronic interface. Each physician anonymously reviews the other physician’s diagnosis and rationale to reconcile initial differences. A third senior physician adjudicates persisting disagreements.

In this nationwide mortality study, we defined causes of death using strict guidelines and coding on the basis of the 10th revision of the International Statistical Classification of Diseases and Related Health Problems (appendix).^7^ Myocardial infarction was defined as an episode of severe chest pain lasting between 30 min and 24 h with at least one additional symptom such as shortness of breath, vomiting, or left arm pain.^10^ Stroke death was defined as a sudden onset of paralysis of one or more limbs in the month preceding death in combination with at least one additional symptom, such as altered speech, loss of sensation, or loss of vision.^10^ Stroke could not be further categorised by subtype. These criteria are consistent with the international case definitions that endorse the use of verbal autopsy to classify cardiovascular deaths in settings without complete death registration and certification.^11^ The verbal autopsy yields results comparable to in-hospital vascular deaths.^10^ 4043 (5%) of 84 625 deaths that occurred in 2002–03 were randomly reinvestigated by an independent team in 2004, and the results were highly consistent.^9^

### Statistical analysis

We determined the age-specific proportion of deaths due to ischaemic heart disease and stroke among men and women for each year for 2001–13, weighted by the sampling probability of the rural and urban population for each state. We smoothed these proportions using 3-year centred moving averages and extrapolated to 2000 and 2014–15 using procedures described earlier.^12^ To derive age-standardised mortality rates for ischaemic heart disease and stroke, we applied these proportions to the national mortality rates reported by the UN Population Division (2000–15)^13^ and standardised rates to the WHO standard population.^14^ We then determined age-standardised mortality rates for rural, urban, and state populations by partitioning the national death totals using Sample Registration System vital statistics.^8^ We defined adult men and women as those aged 15 years or older. We excluded a small proportion of children younger than 15 years who died from cardiovascular disease.

Because of the large sample size of the Million Death Study, uncertainty is mostly attributable to cause of death assignment rather than to random error.^9^ We therefore estimated the influence of coding differences by assigning lower and upper bounds for estimates of mortality rate. The lower bound included deaths initially assigned to the same category by both physicians, whereas the upper bound included deaths initially assigned to a category by either one or both physicians. To estimate the effect of ill-defined causes of death (appendix), we redistributed ill-defined deaths proportionally to other causes for each 5-year age group. We derived average age-standardised annual mortality rates at ages 30–69 years by state among men and women for 2001–04 and 2011–13 and examined the association using linear correlation, weighted by population size. Because stroke mortality varied considerably across states, we separately analysed the subgroup of high-burden states (ie, those with high stroke mortality); all other states were classified as low burden (appendix). We then estimated the annual percentage change of age-standardised and age-specific mortality rates for 2001–15 using the age-period-cohort method (appendix). We computed rate ratios of age-specific mortality rates in each 5-year birth cohort and 5-year calendar period relative to the median birth cohort (1961–65) and calendar period (2005–10), respectively. We used SAS version 9.4 and R version 3.3.2 for analyses, and ArcMap version 10.5 for maps.

### Role of the funding source

The funding sources had no role in the study design, data collection, data analysis, data interpretation, or writing of the manuscript. The corresponding author had full access to all the data and had final responsibility for the decision to submit for publication.

## Results

**Figure 1:**
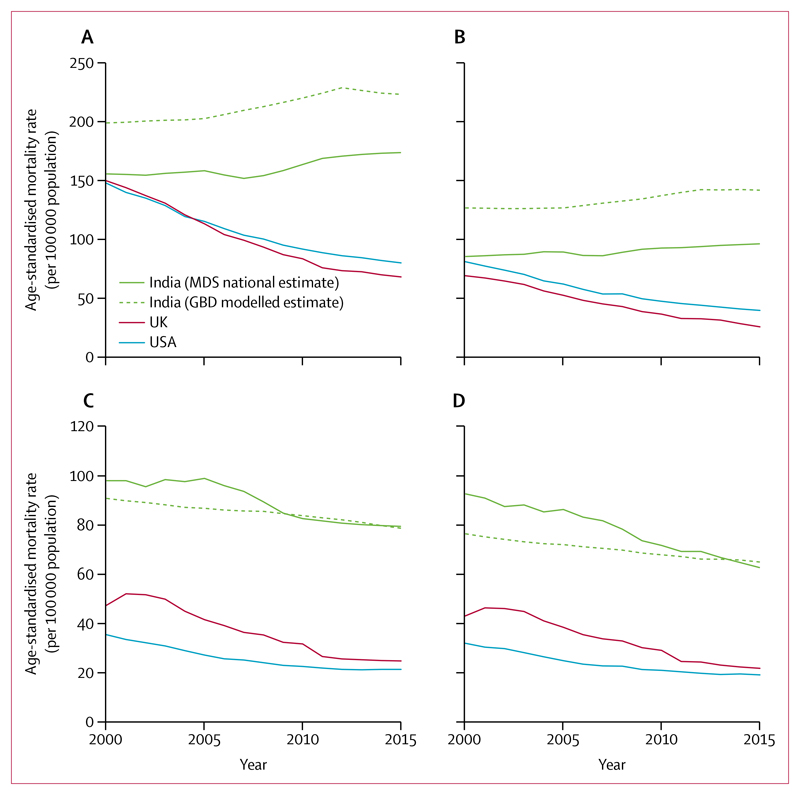
Secular trends in age-standardised mortality rates among men and women (aged ≥15 years), 2000–15 Ischaemic heart disease in men (A) and women (B), and stroke in men (C) and women (D). All rates are standardised to the WHO population. Ischaemic heart disease and stroke deaths under age 15 years are rare and were excluded from MDS and GBD rates to ensure comparability. MDS=Million Death Study. GBD=Global Burden of Disease. Data for the UK and the USA are from the WHO Mortality Database;^15^ we forecasted these rates to 2015 for comparability.

We examined 472 113 deaths of individuals older than 15 years from 2001 to 2013, including 111 977 deaths due to cardiovascular disease (68 904 [61·5%] adult men and 43 073 [38·5%] women; appendix). Cardiovascular disease caused more than 2·1 million deaths in India in 2015 at all ages, or over a quarter of all deaths (table 1). Total cardiovascular deaths comprised 1·3 million cardiovascular deaths at ages 30–69 years: 0·9 million (68·4%) from ischaemic heart disease, 0·4 million (28·0%) from stroke, and 47 000 (3·5%) from rheumatic heart diseases and other cardiovascular diseases. Between 2000 and 2015, the national age-standardised mortality rate from ischaemic heart disease increased among men and women (figure 1, table 2), and the pattern was similar in most states. The highest rates in 2015 were in Andhra Pradesh, Punjab, and Tamil Nadu. In 2000, the age-standardised mortality rate of ischaemic heart disease at all ages in India was similar to high-income countries such as the UK. By 2015, in India these mortality rates rose to more than double those of the UK or USA.

By contrast, the age-standardised mortality rates at all ages for stroke fell among men and women during the 15 years. The USA and UK showed similar downward trends in age-standardised stroke mortality at all ages, but the rates in India remained about three times higher throughout.

**Figure 2:**
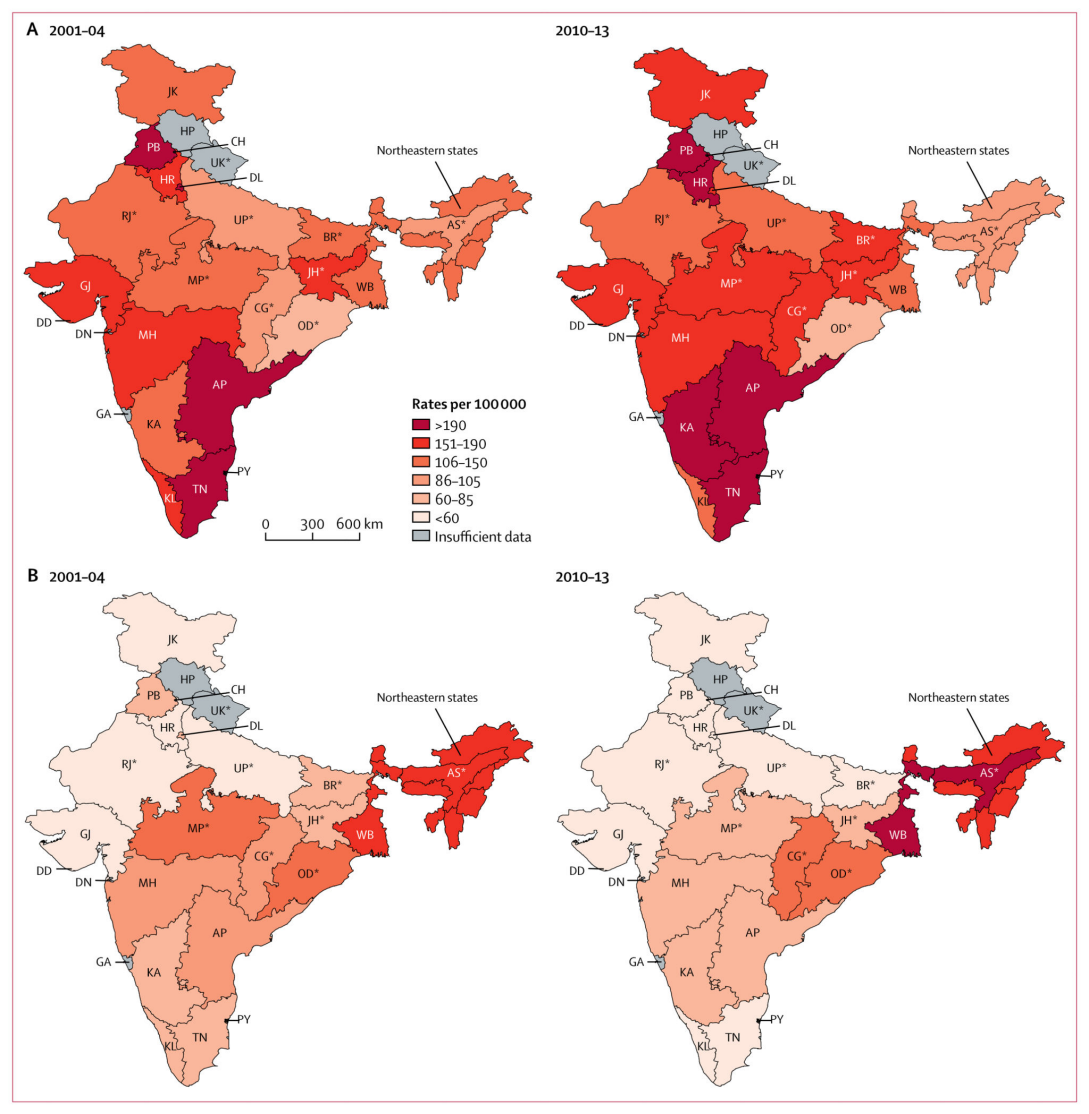
Geographical distribution of cause-specific mortality rate among men and women aged 30–69 years, 2001–04 and 2010–13 Ischaemic heart disease (A) and stroke (B). All rates are standardised to the WHO population. Northeastern states include Sikkim, Arunachal Pradesh, Nagaland, Manipur, Mizoram, Tripura, and Meghalaya. AP=Andhra Pradesh. AS=Assam. BR=Bihar. CH=Chandigarh. CG=Chhattisgarh. DD=Daman and Diu. DN=Dadra and Nagar Haveli. DL=Delhi. GA=Goa. GJ=Gujarat. HP=Himachal Pradesh. HR=Haryana. JH=Jharkhand. JK=Jammu and Kashmir. KA=Karnataka. KL=Kerala. MH=Maharashtra. MP=Madhya Pradesh. OD=Odisha. PB=Punjab. PY=Pondicherry. RJ=Rajasthan. TN=Tamil Nadu. UK=Uttarakhand. UP=Uttar Pradesh. WB=West Bengal. *Lower-income states, where about half of India’s population reside.

The subsequent analyses focus on mortality at ages 30–69 years as these deaths have less misclassification of causes than deaths at ages 70 years or older,^9^ and because they represent avoidable deaths of greater public health importance.^16^ The geographical distribution of stroke mortality rates varied more than did ischaemic heart disease mortality rates (figure 2). A third of premature stroke deaths occurred in a small cluster of states and union territories representing about a sixth of India’s population. High-burden states for male stroke deaths were Assam, West Bengal, Chhattisgarh, and the northeast states (ie, Sikkim, Arunachal Pradesh, Nagaland, Manipur, Mizoram, Tripura, and Meghalaya), and high-burden states for female stroke deaths were Assam, West Bengal, Odisha, Chhattisgarh, and the northeastern states.

In these states, stroke mortality increased significantly and were about three times higher than the national average. At ages 30–69 years, stroke mortality comprised about three-fifths of all cardiovascular deaths in states with a high burden of stroke versus about a quarter of all cardiovascular deaths in the remaining low-burden states (table 1). The proportion of cardiovascular deaths to all deaths at these ages was higher in the states with a high burden of stroke than in the low-burden states.

**Figure 3:**
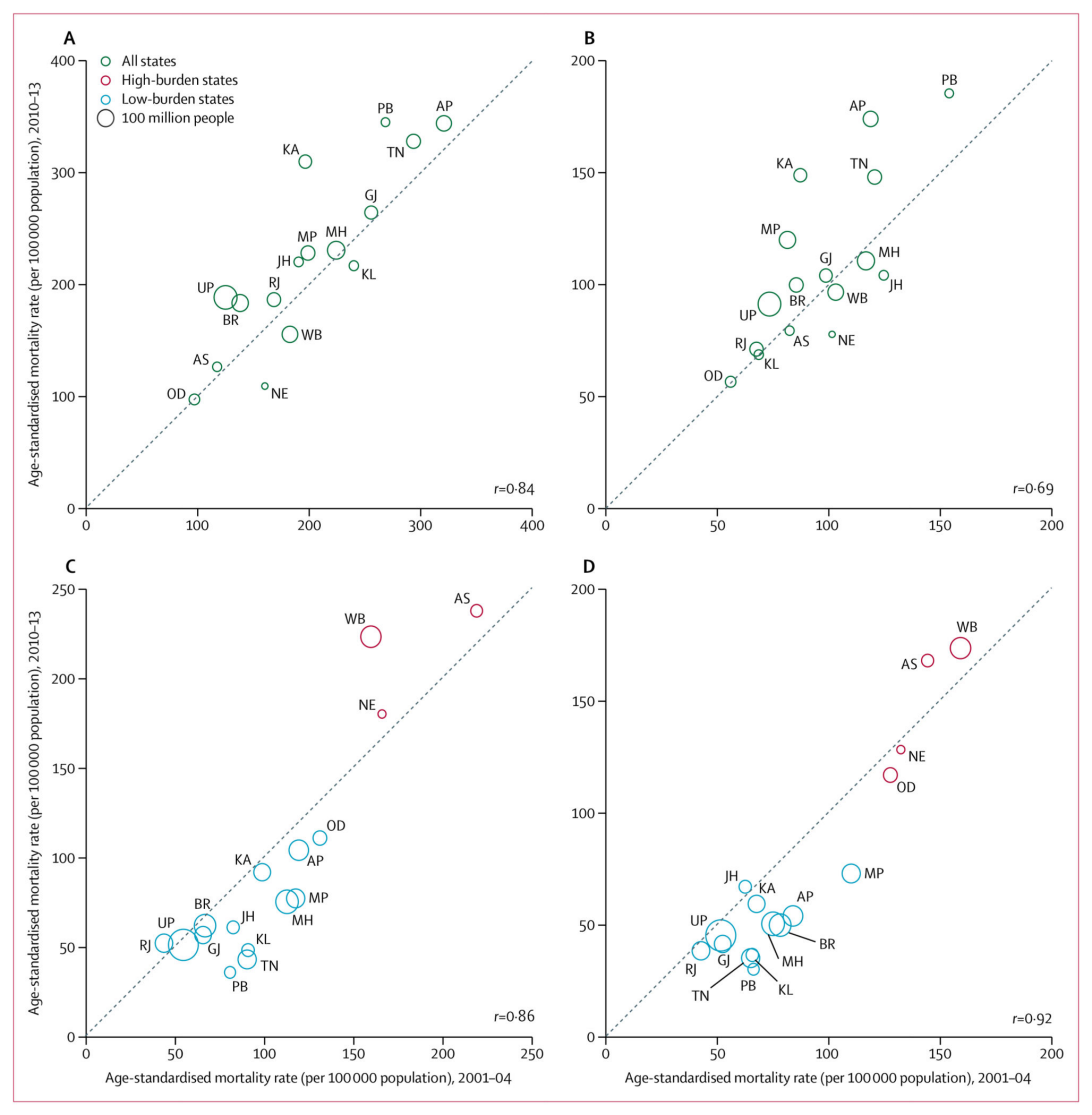
Cause-specific mortality rate in 2010–13 versus 2001–04 among men and women aged 30–69 years, by state Ischaemic heart disease among men (A) and women (B), and stroke among men (C) and women (D). Rates are standardised to the WHO population. The area of each circle is proportional to the state population (2010). The dotted line represents equality, so circles above this line indicate a relative increase and vice versa. AS, BR, JH, MP, OD, RJ, and UP are lower-income states, where about half of India’s population lives. States and union territories with a population of less than 26 million people are suppressed for clarity (appendix). We excluded Himachal Pradesh, Chandigarh, Uttarakhand, Daman and Diu, Dadra and Nagar Haveli, Goa, Lakshadweep, Pondicherry, and Adaman and Nicobar Islands because of sparse data. AP=Andhra Pradesh. AS=Assam. BR=Bihar. GJ=Gujarat. JH=Jharkhand. KA=Karnataka. KL=Kerala. MH=Maharashtra. MP=Madhya Pradesh. NE=Northeastern states (Sikkim, Arunachal Pradesh, Nagaland, Manipur, Mizoram, Tripura, and Meghalaya). OD=Odisha. PB=Punjab. RJ=Rajasthan. TN=Tamil Nadu. UP=Uttar Pradesh. WB=West Bengal.

Stroke mortality rates at ages 30–69 years fell in low-burden states. However, stroke mortality rates increased or did not change substantially in high-burden states in men and women (figure 3). States with high stroke mortality rates had low mortality rates of ischaemic heart disease and vice versa (appendix).

At ages 30–69 years, the probability of dying from ischaemic heart disease (in the absence of other causes) between 2000 and 2015 increased from 10·4% to 13·1% in men and from 4·8 to 6·6% in women. By contrast, the probability of dying from stroke fell marginally from 5·7% to 5·0% in men and 5·0% to 3·9% in women (table 2).

**Figure 4:**
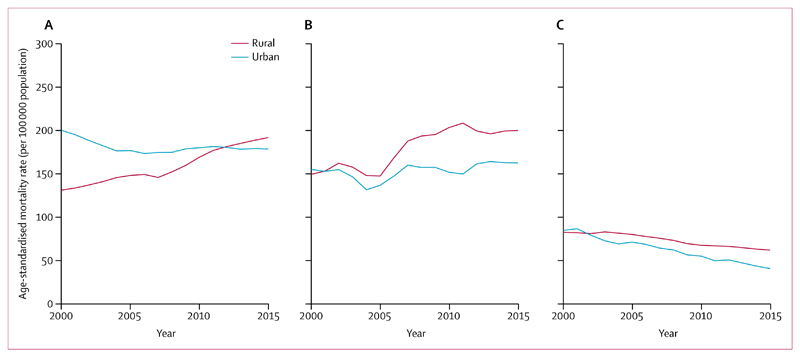
Secular trends in age-standardised mortality rates among men and women aged 30–69 years, by residence, 2000–15 Ischaemic heart disease (A) and stroke in high-burden (B) and low-burden states (C). All rates are standardised to the WHO population.

To further explore these results, we examined rural and urban differences and age-specific trends, and did age-period cohort analyses. Although ischaemic heart disease mortality at ages 30–69 years was lower in rural areas compared with urban areas at the start of the study, rural rates rose rapidly, surpassing urban rates by 2015 in both sexes (figure 4). Stroke mortality at ages 30–69 years in the high-burden states also rose more sharply in rural than in urban areas in both sexes. In states with a low burden of stroke, stroke mortality rates fell more rapidly in urban than rural areas in both sexes.

From 2000 to 2015, age-specific mortality rates increased among middle-aged adults for ischaemic heart disease nationally and for stroke in high-burden states, and age-specific mortality trends for stroke declined in the low-burden states (appendix). Age-period cohort analysis showed that the maximum increase for ischaemic heart disease mortality for men was 2·4% annually (95% CI 2·0–2·8) in those aged 50–54 years and for women was 2·8% (1·6–3·9 in those aged 35–39 years; appendix). The relative risk of ischaemic heart disease mortality increased with each successive birth cohort among men, with the highest increases among the most recent birth cohorts for both sexes (appendix). Compared with women born in the 1960s, women born in the late 1970s had a relative risk of 1·42 (95% CI 1·25–1·60; excess mortality 42%), whereas among men, the relative risk was 1·30 (1·21–1·40; excess mortality 30%). In the high-burden states, age-standardised stroke mortality rates rose by 5·4% (4·8–6·0) per year among men and 1·5% (0·8–2·3) per year among women, peaking at ages 40–49 years. Men born in the 1980s had a relative risk of 3·42 (2·53–4·62). In the low-burden states, age-standardised stroke mortality fell by 1·5% (95% CI 1·0–2·1) per year and 2·7% (2·1–3·4) per year among men and women, respectively, with nadirs at 55–59 years of age. In these states, Indians born after 1960 had about half the risk of dying from stroke versus those born in the 1930s.

**Figure 5:**
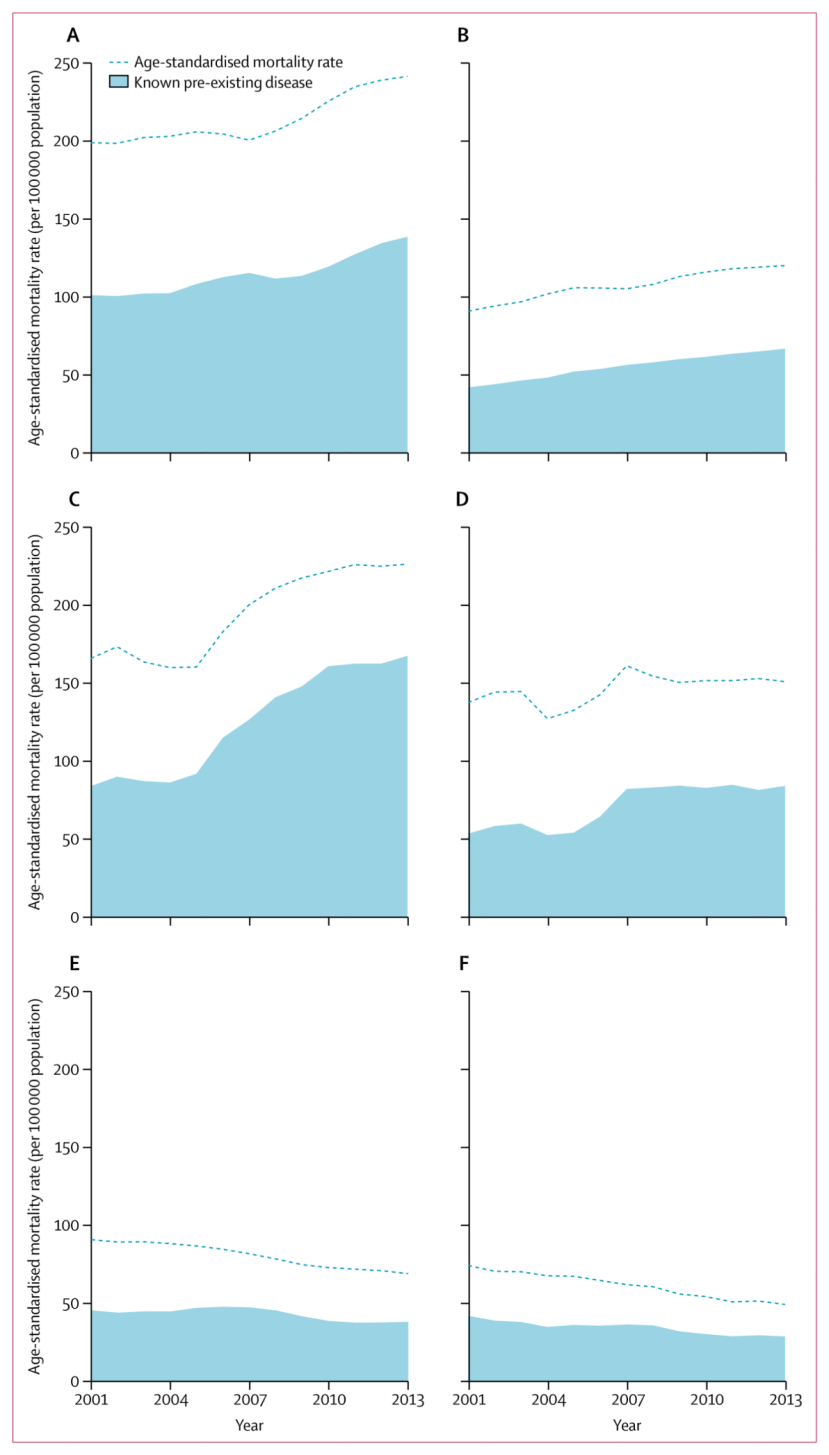
Secular trends in the proportion of men and women aged 30–69 years with known pre-existing disease dying of ischaemic heart disease or stroke, 2001–13 Ischaemic heart disease in men (A) and women (B), and stroke in high-burden states in men (C) and women (D) and low-burden states in men (E) and women (F). The shaded areas represent proportions overlaid onto age-standardised mortality rates (per 100 000 population). All rates are standardised to the WHO population.

The proportion of those dying of ischaemic heart disease with a diagnosis of pre-existing heart disease rose in both sexes between 2001 and 2013 (figure 5, appendix). At least half of these individuals were taking no regular medications (54·1% of men and 50·4% of women in 2013, with 22·2% and 22·8%, respectively, reporting unknown medication status). In high-burden states, the proportion of stroke deaths with a history of pre-existing stroke further increased in both sexes, and the majority of these individuals were taking no medications (56·6% of men and 60·1% of women in 2013, with 23·3% and 24·0%, respectively, reporting unknown medication status). In states with a low burden of stroke, little change occurred in the proportion of individuals who died of stroke and reported a history of stroke, but a substantial portion of those individuals were taking no medications before death (47·7% among men and 48·2% among women in 2013, with 16·1% and 15·4%, respectively, reporting unknown medication status). Missing data were minimal (2·7% for medication status, ≤0·05% for all other variables).

## Discussion

Ischaemic heart disease mortality is rising in India, more in rural than urban areas, with the greatest increases in young adults—especially those born after 1970. Nationally, decreases in stroke deaths were small, but this masks a previously undocumented cluster of high stroke mortality in northeast India with entirely different trends over time than the rest of the country. A large and growing proportion of the ischaemic heart disease and stroke deaths in high-burden states reported earlier diagnosis of cardiovascular disease, but low medication use.

Although mostly documented in high-income countries, established cardiovascular risk factors might account for the urban–rural variations in ischaemic heart disease and stroke, and the comparatively high cardiovascular mortality rates in India. Every 20 mm Hg increase in systolic blood pressure doubles the risk of cardiovascular mortality.^17^ The rural prevalence of hypertension has reportedly increased to match the urban prevalence,^18^ and this increase could be one factor driving the observed urban–rural differences in mortality rates. Smokers have large excesses of fatal and non-fatal ischaemic heart disease and stroke,^19,20^ and men who are illiterate and reside in rural areas are increasingly likely to smoke manufactured cigarettes as opposed to locally grown beedis.^21^ Smoking cessation rates in middle age are far below those of high-income countries.^21^ Hence, although not directly studied here, smoking is a major determinant of the age and region specific variation in cardiovascular disease rates. Although diabetes is increasing more rapidly in urban than rural areas,^22^ the rates of untreated diabetes in rural versus urban areas are still not known. In Mexico, the risks of cardiovascular death from diabetes (which is mostly untreated or undertreated), was much higher than the risks from diabetes in high-income countries (which is mostly treated).^23^ Thus, reduced quality, availability, and accessibility of secondary treatment in rural areas might play an important part in the observed rural–urban disparities in cardiovascular mortality.^24^

The role of other established risk factors in India is less clear. In high-income countries, increased body-mass index (BMI) increases ischaemic heart disease and stroke mortality among obese individuals.^25^ However, evidence from high-income countries might not be generalisable to the unique Indian phenotype of central obesity. A low BMI might paradoxically predict increased mortality in India for unknown reasons.^26^ Further research is required to confirm how obesity and other established risk factors affect cardiovascular mortality in India. Evidence suggests that high-carbohydrate diets increase ischaemic heart disease risk,^27^ although carbohydrate intake has remained relatively stable in rural and urban areas.^2^

Furthermore, the risk factors driving ischaemic heart disease mortality might differ from those affecting stroke in high-burden states. Northeastern states have substantial populations of Sino-Tibetan ethnicity,^22^ and these populations might resemble the Chinese and Mongolian populations in which stroke dominates ischaemic heart disease. The incidence of haemorrhagic stroke appears to be particularly high among the Chinese population,^6^ and hypertension is the primary risk factor for this subtype. Although dietary factors, such as excessive salt intake, might increase hypertension prevalence in northeastern states,^28^ hypertension prevalence varies substantially across high-burden states. Inadequate stroke care might have a role in increasing mortality rates,^6^ and novel risk factors such as endemic infections might also be associated with stroke in these areas.^6^

On current trends, the UN Sustainable Development Goal to reduce cardiovascular mortality rates at ages 30–69 years by a third by 2030 is unlikely to be met without substantial progress in India. Moreover, ischaemic heart disease rates in Mexico and China also rose between 2000 and 2015.^1^ Modelled estimates by the Global Burden of Disease (GBD) for India propose higher ischaemic heart disease mortality but lower stroke mortality than our observed rates.^29^ The GBD has been criticised on the basis of the assumptions required to generate cause-specific cardiovascular mortality rates for more than 70% of global populations for whom no such data exist. Moreover, the GBD reclassifies ill-defined causes of death as ischaemic heart disease. The 10th revision of the International Statistical Classification of Diseases and Related Health Problems code I69—one of the most common stroke codes—is also reclassified into various causes including ischaemic heart disease. This classification might lead to the overestimation of ischaemic heart disease mortality rates and the underestimation of stroke mortality rates (appendix).

Our findings suggest that a large agenda for secondary treatment remains in India. Cheap, generic statins reduce cardiovascular mortality by 15% for every 1 mmol/L of LDL cholesterol lowered.^30^ Although statin sales might be increasing,^31^ our finding of low reported medication use is consistent with previous estimates that less than 10% of Indians with cardiovascular disease use statins regularly. ^31,32^ Effective long-term management of diabetes and hypertension is similarly lacking, particularly in rural areas.^18,33^ Use of the polypill—a combination of generic aspirin, antihypertensive, and statin combined into one pill—is a widely practicable strategy for India.^34^ Smoking cessation rates remain low,^21^ and many quit smoking as a consequence of rather than to avoid cardiovascular disease. However, most health services in India are not set up to capture patients with cardiovascular disease or to manage established risk factors.^35^

This is the first nationally representative study to measure cardiovascular mortality in India. Verbal autopsy remains the only feasible method of defining probable causes of death occurring at home, and without medical attention in most low-income and middle-income countries,^36^ we used this important tool to uncover the most complete picture of longitudinal mortality trends to date. However, some limitations exist. First, clinical autopsy and medical imaging were not available to confirm cause of death or stroke subtype. Nevertheless, the verbal autopsy is a widely used, validated, and reliable instrument for classifying ischaemic heart disease and stroke deaths in India.^9,10^ Further research is required to confirm whether haemorrhagic stroke has decreased in low-burden states as it has in other countries,^37^ and to assess whether increases in ischaemic heart disease mortality might preclude the occurrence of stroke mortality as a competing risk. In our data, only a minority of individuals who died of ischaemic heart disease reported previous stroke (appendix). Second, we had only crude data on medication use and were unable to access medical records to verify whether the drugs used were for cardiovascular disease, which would suggest that the extent of secondary treatment is quite low in India.^30,35^ Further research is required to better characterise the use of secondary prevention among those with cardiovascular disease.

This study has shown that cardiovascular mortality in India shows unexpected patterns that have not been well characterised previously. In less than two decades, ischaemic heart disease mortality in rural India has surged and surpassed urban levels, whereas stroke mortality has diverged across geographical areas. The National Rural Health Mission and the National Program for Prevention and Control of Cancer, Diabetes, Cardiovascular Diseases and Stroke play a vital part in improving risk factor management and secondary prevention to reduce cardiovascular mortality. Improvement and sustaining of these programmes are needed to collect local evidence that will lead to the understanding of how established and novel risk factors operate in India to produce these distinctive epidemiological patterns.

## Supplementary Material

Appendix

## Figures and Tables

**Table 1:** National and subnational cardiovascular death estimates for 2015 from the Million Death Study

	Male/female population in 2015 (millions)	Ischaemic heart disease deaths (thousands)		Stroke deaths (thousands)		Other cardiovascular deaths (thousands)		All cardiovascular deaths/all-cause deaths (thousands)	
				
		Men	Women	Men	Women	Men	Women	Men	Women
**All ages**									

Study deaths, 2001–13	··	44	22	23	18	3	2	69/270	43/202
National	680/632	878	488	374	314	54	51	1306/4487	852/3650

**Age 30−69 years**									

National	274/260	613	295	218	157	26	22	857/2501	473/1653
High-burden stroke states	38/45	52	32	76	64	9	6	137/345	103/294
Low-burden stroke states	236/215	561	262	142	93	17	15	720/2156	370/1358
Rural areas	180/174	424	206	150	116	15	14	589/1844	337/1255
Urban areas	94/87	189	88	68	40	10	7	268/657	136/398

Numbers of cause-specific deaths were generated from study estimates. Subnational totals were adjusted to match national totals. Totals might not add up exactly because of rounding. Deaths in children younger than 15 years are excluded from totals because cardiovascular deaths are rare in this age group.

**Table 2:** Age-standardised mortality rates for ischaemic heart disease and stroke (per 100 000 person years) for 2000–15 among men and women (aged ≥15 years)

	Ischaemic heart disease				Stroke			
	Men		Women		Men		Women	
	2000	2015	2000	2015	2000	2015	2000	2015
**All ages**								

National (lower, upper bounds*)	155 (110, 179)	173 (153, 195)	85 (55, 104)	96 (81, 109)	98 (73, 101)	80 (66, 82)	93 (71, 97)	63 (53, 65)

**Age 30–69 years**								

Period risk†	10·4%	13·1%	4·8%	6·6%	5·7%	5·0%	5·0%	3·9%
National	199	245	88	121	102	88	85	66
High-burden stroke states	156	160	78	85	161	227	137	157
Low-burden stroke states	208	262	90	135	92	66	76	45
Rural areas	181	255	81	127	102	91	85	71
Urban areas	283	234	116	123	110	88	87	55

All rates are weighted by sampling probability and standardised to the WHO population.

* Bounds are defined as estimates including only deaths immediately assigned to the same cause of death category by two physicians (lower bound), and deaths immediately assigned to the cause of death category by only one physician (upper bound).

† Period risk is the probability of cause-specific death if no other causes of death occurred. The age-specific period risk is calculated by multiplying the mortality rate by the duration of the age range. The period risk for ages 30–69 years is the cumulative total of the age-specific period risks.

## Research in context

### Evidence before this study

We searched MEDLINE and Embase for articles on the adult population of India published from Jan 1, 2000, to Dec 6, 2017, using the search terms “exp India/ep” and “exp Cardiovascular Diseases/mo” in MEDLINE, and “exp India/ep”, “exp cardiovascular disease/”, and “exp mortality/ or exp premature mortality/” in Embase, with no language restrictions. We identified 18 studies measuring cardiovascular mortality, most of which were hospital based and none of which were nationally representative.

### Added value of this study

This is the first nationally representative study to measure cardiovascular mortality in India, providing the most complete picture of longitudinal mortality trends to date. In less than two decades, ischaemic heart disease mortality in rural India has surged and surpassed that of urban areas, whereas stroke mortality has diverged across geographical areas.

Stroke mortality is increasing in a small cluster of high-burden states in the northeastern region of India, representing a sixth of the country’s population, yet accounting for a third of total stroke deaths. We uncovered a large secondary prevention target for ischaemic heart disease and stroke: most deaths were among individuals with previously known cardiovascular disease, and at least half were not taking any regular medications.

### Implications of all the available evidence

Cardiovascular mortality in India shows unexpected patterns that have not been well characterised previously. Despite uncertainties about the causes that account for the marked divergence between ischaemic heart disease and stroke, and between stroke patterns in different regions, a large proportion of Indians with pre-existing cardiovascular disease are presently untreated and would benefit from secondary prevention and smoking cessation. Without progress in India against cardiovascular disease, the Sustainable Development Goals to reduce cardiovascular disease and non-communicable disease mortality by 2030 will be difficult to achieve.
