# Supplementary material for: Divergent trends in ischaemic heart disease and stroke mortality in India from 2000 to 2015: a nationally representative mortality study
Source: Lancet Glob Health. Author manuscript; Available in PMC 2020 Jan 4. (PMC6942542; doi:10.1016/S2214-109X(18)30242-0)
Supplement: Appendix [file EMS85331-supplement-Appendix.pdf]

THE LANCET
Global Health

Supplementary appendix

This appendix formed part of the original submission and has been peer reviewed. We post it as supplied by the authors.

Supplement to: Ke C, Gupta R, Xavier D, et al.Divergent trends in ischaemic heart disease and stroke mortality in India from 2000 to 2015: a nationally representative mortality study. *Lancet Glob Health* 2018; **6:** e914–23.

Divergent trends in ischaemic heart disease and stroke mortality in India from 2000 to 2015: a nationally representative mortality study

Million Death Study Collaborators

Shazia Allarakha*, Dharmappa Basavarajappa*, Vikas Dhimar*, Harsh Jaiswal*, Shrikant Kalaska*, Geetha Memon*, Saritha Nair, Vishnu Rao*, Reeta Rasaily, Damodar Sahu, Shweta Sharma*, Deepak Kumar Shukla*, Jitenkumar K. Singh, Lucky Singh, Anju Sinha, Indian Council of Medical Research, Ansari Nagar, New Delhi, India

Shally Awasthi, Department of Pediatrics, Institute of Clinical Epidemiology, King George Medical College, Chatrapati Shahu Ji Maharaj Medical University, Lucknow, Uttar Pradesh, India

Jayant K. Banthia (retired), Government of Maharashtra, Mumbai, Maharashtra, India

Rajesh Dikshit, Tata Memorial Hospital, Mumbai, Maharashtra, India

Rajesh Kumar, J.S. Thakur, School of Public Health, Post Graduate Institute of Medical Education and Research, Chandigarh, India

Faujdar Ram (retired), Usha Ram, Department of Public Health and Mortality Studies, International Institute for Population Sciences, Deonar Mumbai, Maharashtra, India

Jay Sheth, Department of Community Medicine, Ahmedabad Municipal Corporation's Medical Education Trust Medical College, Ahmedabad, Gujarat, India

George D'Souza, Rehana Begum*, Denis Xavier*, St. John's Medical College and Research Institute, Bangalore, India

Rajeev Gupta*, Academic Research Development Unit, Rajasthan University of Health Sciences, Jaipur, Rajasthan, India

Hellen Gelband*, Prabhat Jha*, Calvin Ke*, Patrycja Kolpak, Wilson Suraweera, Centre for Global Health Research, St. Michael's Hospital, Dalla Lana School of Public Health, University of Toronto, Toronto, ON, Canada

Yogeshwar V. Kalkonde*, Society for Education, Action and Research in Community Health, Gadchiroli, Maharashtra, India

Prashant Mathur*, National Centre for Diseases Informatics & Research, Indian Council of Medical Research, Bangalore, Karnataka, India

Dorairaj Prabhakaran*, Public Health Foundation of India, Gurugram, Haryana, India

***ICMR-CGHR Vascular Disease Working Group**

Appendix I. Supplementary Methods

Verbal Autopsy

Verbal autopsy (VA) is a widely-used instrument for classifying causes of death in low- and middle-income countries such as India, where most deaths occur at home without medical attention. Although the gold standard for establishing cause of death (i.e. autopsy) rarely exists in this setting, the VA has been validated and shown to be highly reliable for classifying ischemic heart disease (IHD) and stroke deaths (test-retest odds ratio of 1·0, 95% confidence interval 0·9–1·2).[1] For the portion of deaths occurring in a hospital setting, the VA performs well for identifying cardiovascular causes of death. An independent validation study in India found VA to have an excellent specificity (92·7–95·0%) and reasonable sensitivity (75·0%) for IHD and stroke deaths compared to hospital records.[2] It is known that after the 70 years of age, there is an increased fraction of deaths where the cause is unclassifiable by VA.[1] To improve sensitivity, we therefore focused on premature deaths from ages 30 to 69 years.

Cardiovascular causes of death were categorized as shown below (Table S1). All causes of death in our study were represented as 3-digit codes from the *International Statistical Classification of Diseases and Related Health Problems* version 10 (ICD-10).

Supplementary Table S1 Cardiovascular causes of death used in this study compared to the Global Burden of Disease studies[3]

Cause of Death Category	Present Study ICD-10 Codes	Global Burden of Disease (GBD) ICD-10 Codes	Remarks
Ischemic heart disease	I20–I25, I46, R55, R96	I20–I25·9	We included I46 (cardiac arrest) based on local coding conventions. We included R55 (syncope and collapse) and R96 (other sudden death, cause unknown) as we judged that most of these cases would presumably involve myocardial infarction. In practice, these adjustments made little absolute difference because the vast majority of premature mortality in this category were classified under I21 (88·9%), I22 (3·9%), and I25 (3·0%).
Stroke	G45–G46, I60–I67, I69, G81–83	G45–G46·8, I60–I61·9, I62·0–I62·03, I63–I63·9, I65–I66·9, I67·0–I67·3, I67·5–I67·7, I69·0–I69·198, I69·20–I69·398	We included I64 (stroke, not specified as hemorrhage or infarction) because due to the nature of verbal autopsy, the majority of stroke deaths in our study were in this category. We included codes for hemiplegia and other paralytic syndromes (G81–G83) based on local coding conventions. In practice, this adjustment made little absolute difference because the vast majority of deaths in this category were classified under I64 (72·9%), I69 (17·1%), and I61 (6·5%).
Rheumatic heart disease	I00–I02, I05–I09, I38	I01–I01·9, I02·0, I05–I09·9	I38 (endocarditis, valve unspecified) was included as we judged that in our population, this would most commonly be secondary to rheumatic heart disease.
Other cardiovascular	I11,I28,I30–I31,I34–I37,	I11–I11·9, I28–I28·8, I30–I31·1, I31·8, I31·9, I33–	We included I50 (heart failure) in this category even though it is not classified in

disease†	I40–I42,I47–I48,I51,I70–I78,I80–I83,I86–I89, R00, R01, R03	I38·9, I39–I42·9, I47–I48·92, I51·0–I51·6, I70·2–I70·8, I71–I71·9, I72–I78·9, I80–I83·93, I86–I89·9, I91·9	this category under the GBD report. Endocarditis is excluded from this category as noted above. We decided to include R00 (abnormalities of heart beat), R01 (cardiac murmurs and other cardiac sounds), and R03 (abnormal blood-pressure reading without diagnosis) based on local coding conventions.

†Includes hypertensive heart disease, cardiomyopathy and myocarditis, atrial fibrillation and flutter, aortic aneurysm, peripheral vascular disease, heart failure, and other cardiovascular and circulatory diseases

Period Risk

Period risk is the probability of cause-specific death if there were no other causes of death. The age-specific period risk is calculated by multiplying the mortality rate by the duration of the age range. The period risk for ages 30–69 years is the cumulative total of the age-specific period risks.

Identifying States with Higher Burden of Stroke

Within the distribution of premature stroke mortality across India, there was a geographic cluster of states in the eastern and northeastern region with distinctly high rates of age-standardized stroke mortality. We used the "average linkage" algorithm in SAS version 9·4 (SAS Institute, Cary, North Carolina) to formally identify the states included in within this cluster. Based on simulation studies, this is the algorithm of choice when the number of points in each cluster is not similar.[4] We identified the clusters independently for men and women, accounting for the most recent age-standardized mortality rates (2010–13 period) and the change in these rates over time (2001–13). We classified this cluster of states with higher stroke rates as "high-burden" states, and all other states as "low-burden" states.

The high-burden states for men were Assam, West Bengal, Chhattisgarh, and northeast states (Sikkim, Arunachal Pradesh, Nagaland, Manipur, Mizoram, Tripura, Meghalaya). For women, the high-burden states were Assam, West Bengal, Odisha, Chhattisgarh, and northeast states.

Age-Period-Cohort Models

Age-period-cohort (APC) models are a method of analyzing mortality rates over time. These models distinguish effects due to aging (due to natural history), period (environmental changes affecting the entire population simultaneously), and birth cohort (changes affecting individuals born in the same time period).[5] Such models have been applied to similar mortality studies in other countries.[6]

Detailed computational descriptions of these models are available elsewhere.[5,7,8] Essentially, the APC model assumes that expected mortality rates can be expressed as a log-linear model with additive effects of age, period, and cohort.[9] Due to the inherent collinearity among these terms, age, period, and cohort deviation parameters are constrained for identifiability. Each parameter is then identified by maximizing the Poisson log likelihood. We examined each 5-year birth cohort from 1936 to 1981 over 5-year calendar periods from 2001 to 2015.

References

1 Aleksandrowicz L, Malhotra V, Dikshit R, *et al.* Performance criteria for verbal autopsy-based systems to estimate national causes of death: development and application to the Indian Million Death Study. *BMC Med* 2014; **12**: 21.

2 Kumar R, Thakur JS, Rao BT, Singh MMC, Bhatia SPS. Validity of verbal autopsy in determining causes of adult deaths. *Indian J Public Health* 2006; **50**: 90–4.

3 Wang H, Naghavi M, Allen C, *et al.* Global, regional, and national life expectancy, all-cause mortality, and cause-specific mortality for 249 causes of death, 1980–2015: a systematic analysis for the Global Burden of Disease Study 2015. *The Lancet* 2016; **388**: 1459–544.

4 Kuiper FK, Fisher L. 391: A Monte Carlo Comparison of Six Clustering Procedures. *Biometrics* 1975; **31**: 777–83.

5 Holford TR. Understanding the Effects of Age, Period, and Cohort on Incidence and Mortality Rates. *Annu Rev Public Health* 1991; **12**: 425–57.

6 Shiels MS, Chernyavskiy P, Anderson WF, *et al.* Trends in premature mortality in the USA by sex, race, and ethnicity from 1999 to 2014: an analysis of death certificate data. *The Lancet* 2017; **389**: 1043–54.

7 Rosenberg PS, Anderson WF. Age-Period-Cohort Models in Cancer Surveillance Research: Ready for Prime Time? *Cancer Epidemiol Prev Biomark* 2011; **20**: 1263–8.

8 Rosenberg PS, Check DP, Anderson WF. A Web Tool for Age–Period–Cohort Analysis of Cancer Incidence and Mortality Rates. *Cancer Epidemiol Prev Biomark* 2014; **23**: 2296–302.

9 Rosenberg PS, Anderson WF. Proportional hazards models and age–period–cohort analysis of cancer rates. *Stat Med* 2010; **29**: 1228–38.

Appendix II. Additional Tables and Figures

Supplementary Table S2 Cardiovascular (CV) deaths among Indians in the Million Death Study from 2001-2013

Study Deaths (male and female), 2001–2013									
	IHD		Stroke		RHD		Other CV disease		All Deaths
Age group (years)	Attributed Deaths	Immediate Agreement†	Attributed Deaths	Immediate Agreement†	Attributed Deaths	Immediate Agreement†	Attributed Deaths	Immediate Agreement†	
15-29	1852	1406	636	405	208	97	235	78	40086
30-44	6624	5606	2022	1561	175	80	333	102	53024
45-59	17402	14907	7548	6105	119	46	760	225	88522
60-69	17963	14509	11639	9510	83	29	1020	308	101521
≥70	21902	16089	19381	15184	92	26	1983	591	188960
Subtotal, age 30-69 years (bounds‡)	41989 (35022, 47265)		21209 (17176, 21947)		377 (155, 468)		2113 (635, 2282)		243067
Rural, age 30-69 years (%)	69·8		78·3		76·7		69·1		
Died in a health facility, age 30-69 years (%)	33·0		29·2		49·0		38·1		
Male Study Deaths, 2001-2013									
	IHD		Stroke		RHD		Other CV disease		All Male Deaths
Age group (years)	Attributed Deaths	Immediate Agreement†	Attributed Deaths	Immediate Agreement†	Attributed Deaths	Immediate Agreement†	Attributed Deaths	Immediate Agreement†	
15-29	1137	878	346	212	103	41	101	28	21953
30-44	4975	4259	1264	969	65	30	155	42	34732
45-59	12647	10952	4642	3756	55	19	431	120	57162
60-69	11766	9635	6584	5358	40	16	560	169	58703
≥70	13145	9940	9803	7747	41	9	1044	310	97289
Subtotal, age 30-69 years (bounds‡)	29388 (24846, 32621)		12490 (10083, 12878)		160 (65, 206)		1146 (331, 1190)		150597
Rural, age 30-69 years (%)	69·5		77·8		80·6		67·7		
Died in a health facility, age 30-69 years (%)	34·5		33·1		56·9		41·8		

Female Study Deaths, 2001-2013									
	IHD		Stroke		RHD		Other CV disease		All Female deaths
Age group (years)	Attributed Deaths	Immediate Agreement†	Attributed Deaths	Immediate Agreement†	Attributed Deaths	Immediate Agreement†	Attributed Deaths	Immediate Agreement†	
15-29	715	528	290	193	105	56	134	50	18133
30-44	1649	1347	758	592	110	50	178	60	18292
45-59	4755	3955	2906	2349	64	27	329	105	31360
60-69	6197	4874	5055	4152	43	13	460	139	42818
≥70	8757	6149	9578	7437	51	17	939	281	91671
Subtotal, age 30-69 years (bounds‡)	12601 (10176, 14644)		8719 (7093, 9069)		217 (90, 262)		967 (304, 1092)		92470
Rural, age 30-69 years (%)	70·3		79·2		73·7		70·8		
Died in a health facility, age 30-69 years (%)	29·6		24·0		43·7		33·9		

Abbreviations: IHD, ischemic heart disease; RHD, rheumatic heart disease

*Includes hypertensive heart disease, cardiomyopathy and myocarditis, atrial fibrillation and flutter, aortic aneurysm, peripheral vascular disease, heart failure, and other cardiovascular and circulatory diseases. †Indicates number of deaths immediately assigned to the same cause of death category by 2 physicians.
‡The lower bound only includes deaths initially assigned to the same category by both physicians. The upper bound includes deaths initially assigned to a category by either one or both physicians.

Supplementary Table S3 Age-specific mortality rates for ischemic heart disease and stroke per 100 000 population from 2001–2004 and 2010–2013 among men and women. All rates are weighted by sampling probability.

	Ischemic heart disease				Stroke			
	Male		Female		Male		Female	
	2001–2004	2010–2013	2001–2004	2010–2013	2001–2004	2010–2013	2001–2004	2010–2013
Age-Specific Mortality by Age Group (years)								
15–19	3·1	3·6	4·1	5·1	2·2	1·5	1·6	1·6
20–24	8·9	8·9	7·9	6·6	2·6	3·1	4·4	2·8
25–29	15·8	18·7	10·4	11·1	4·7	3·4	4·6	3·5
30–34	33·2	34·9	12·8	15·0	7·3	6·9	6·5	4·9
35–39	47·9	60·8	17·4	24·6	11·7	16·3	11·3	10·9
40–44	84·7	105·8	28·4	44·5	22·2	26·3	18·7	17·5
45–49	133·8	170·2	54·5	71·4	44·6	45·0	32·5	36·9
50–54	226·1	281·8	94·9	113·1	84·6	93·1	67·4	62·9
55–59	356·6	408·3	175·9	202·2	169·8	148·0	137·4	106·8
60–64	525·4	599·8	263·6	318·9	312·8	259·5	250·1	215·1
65–69	714·6	804·8	397·7	491·2	479·6	426·0	437·8	340·0
70–74	904·5	962·7	595·8	589·8	688·5	588·5	691·4	492·4
75–79	1216·6	1152·3	874·7	780·7	998·4	801·3	919·8	753·1
≥80	1728·3	1530·7	1134·6	935·1	1498·9	1001·6	1418·7	997·9

Supplementary Table S4 Age-standardized mortality rates (ASMR, per 100 000 population) and proportionate mortality (%) for ischemic heart disease and stroke by sex, state and period for ages 30–69 years. Age-standardized rates utilize the World Health Organization standard population.

	Ischemic heart disease								Stroke							
State	Male				Female				Male				Female			
	ASMR		Proportionate Mortality		ASMR		Proportionate Mortality		ASMR		Proportionate Mortality		ASMR		Proportionate Mortality	
	2001–04	2010–13	2001–04	2010–13	2001–04	2010–13	2001–04	2010–13	2001–04	2010–13	2001–04	2010–13	2001–04	2010–13	2001–04	2010–13
Jammu & Kashmir	147·6	182·6	22·0	31·2	73·6	148·1	13·6	29·4	64·3	54·0	9·6	9·2	54·3	56·9	10·0	11·3
Punjab	268·1	345·0	30·3	38·8	153·9	185·3	25·1	33·3	81·0	35·6	9·2	4·0	66·2	29·9	10·8	5·4
Haryana	239·7	326·3	25·5	33·8	69·2	123·8	12·4	23·1	68·5	49·7	7·3	5·2	44·4	35·3	8·0	6·6
Delhi	271·4	224·6	40·4	34·1	136·7	143·8	24·4	33·4	69·1	47·9	10·3	7·3	79·7	31·2	14·2	7·2
Rajasthan	168·1	186·4	19·3	22·1	67·5	71·1	11·3	13·7	44·0	51·9	5·0	6·2	42·6	38·1	7·1	7·3
Uttar Pradesh	124·7	188·5	11·7	17·0	73·4	91·2	8·0	11·5	54·9	51·0	5·1	4·6	51·5	45·0	5·6	5·6
Bihar	137·9	183·4	15·7	22·7	81·5	119·9	9·6	16·6	67·0	61·6	7·6	7·6	78·2	49·6	9·3	6·8
Assam	117·3	126·6	9·8	11·7	82·5	79·4	7·8	9·4	219·0	237·1	18·3	21·9	144·4	167·5	13·6	19·8
West Bengal	182·6	155·5	21·3	19·5	103·1	96·6	14·5	16·1	159·9	222·7	18·7	27·9	159·1	173·0	22·3	28·8
Jharkhand	190·4	220·3	16·8	22·9	124·7	104·1	12·8	11·9	82·7	60·7	7·3	6·3	62·5	66·7	6·4	7·6
Odisha	96·8	97·4	8·4	9·9	56·0	56·6	5·8	7·0	131·3	110·5	11·4	11·3	127·6	116·5	13·3	14·4
Chhattisgarh	126·2	196·2	15·3	18·4	76·8	101·6	9·9	12·7	109·9	171·6	13·3	16·1	94·3	98·0	12·2	12·3

Madhya Pradesh	198·7	228·1	17·9	23·0	85·4	99·8	9·6	14·1	117·7	76·9	10·6	7·7	110·1	72·6	12·4	10·3
Gujarat	255·4	264·2	24·1	30·3	98·8	104·0	15·2	18·0	65·9	56·3	6·2	6·5	52·4	41·1	8·1	7·1
Maharashtra	223·9	230·6	22·1	27·2	116·7	110·6	16·0	18·8	113·0	75·0	11·2	8·8	75·0	50·1	10·3	8·5
Andhra Pradesh	320·5	343·9	27·7	31·3	118·8	173·9	16·0	22·8	119·5	103·7	10·3	9·4	83·9	53·7	11·3	7·0
Karnataka	196·3	309·7	19·3	30·7	87·3	148·8	13·3	21·6	99·0	91·4	9·7	9·1	67·6	59·0	10·3	8·6
Kerala	239·7	216·9	27·3	27·1	68·6	68·7	15·1	17·8	91·1	48·0	10·4	6·0	65·7	36·2	14·5	9·4
Tamil Nadu	293·4	327·9	26·7	35·5	120·6	148·0	15·8	23·5	90·6	42·8	8·2	4·6	64·9	34·9	8·5	5·5
Northeast States	160·0	109·3	13·3	10·3	101·5	77·7	9·7	9·3	166·1	179·6	13·8	16·9	132·4	127·8	12·6	15·3

Supplementary Table S5 Percentage of men and women aged 30–69 years with pre-existing disease dying of ischemic heart disease (IHD) and stroke for 2001–13. Rates for men and women were broadly similar and combined in this table. All values are percentages.

State	Previous Heart Disease Among IHD deaths		Previous Stroke Among Stroke Deaths	
	Yes	Unknown	Yes	Unknown
Jammu & Kashmir	63.3	6.4	31.7	4.8
Punjab	65.9	1.8	33.6	3.1
Haryana	51.3	3.1	52.8	5.0
Delhi	45.3	28.3	40.7	24.4
Rajasthan	61.7	4.5	67.1	5.4
Uttar Pradesh	61.0	3.5	62.2	2.1
Bihar	56.7	6.0	57.5	2.5
Assam	52.3	6.6	57.3	2.6
West Bengal	50.7	12.6	64.5	5.9
Jharkhand	52.1	7.8	62.8	4.1
Orissa	49.0	14.6	13.7	17.8
Chhattisgarh	50.7	4.1	73.5	1.8
Madhya Pradesh	54.1	5.9	68.3	4.7
Gujarat	56.7	3.6	63.4	1.9
Maharashtra	52.5	4.6	71.3	1.7
Andhra Pradesh	58.3	3.0	41.8	6.2
Karnataka	63.0	1.6	76.6	1.3
Kerala	58.0	3.9	51.1	3.4
Tamil Nadu	57.6	6.7	47.3	7.6
Northeast States	45.6	9.9	64.0	4.6
High-Burden Stroke States†			58.9	6.1
All India	56.5	5.6	55.7	5.2

†High-burden states for male stroke: Assam, West Bengal, Chhattisgarh, and northeast states (Sikkim, Arunachal Pradesh, Nagaland, Manipur, Mizoram, Tripura, Meghalaya)
High-burden states for female stroke: Assam, West Bengal, Odisha, Chhattisgarh, and northeast states (Sikkim, Arunachal Pradesh, Nagaland, Manipur, Mizoram, Tripura, Meghalaya)

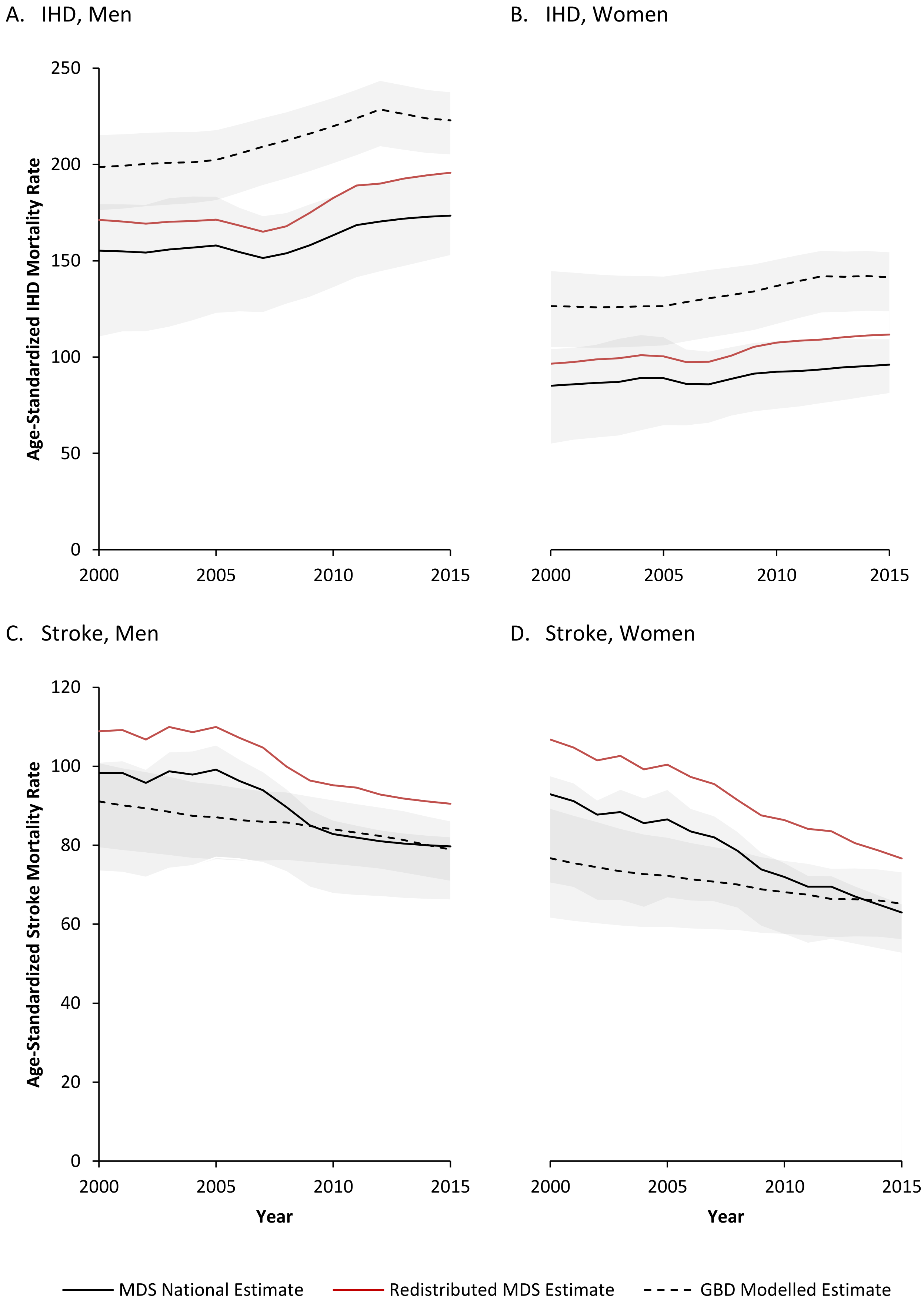

A. IHD, Men
B. IHD, Women
C. Stroke, Men
D. Stroke, Women
Age-Standardized IHD Mortality Rate
Age-Standardized Stroke Mortality Rate
250
200
150
100
50
0
120
100
80
60
40
20
2000
2005
2010
2015
Year
MDS National Estimate
Redistributed MDS Estimate
GBD Modelled Estimate

Supplementary Figure S1 (previous page) Secular trends in age-standardized mortality rates per 100 000 population by sex from 2001 to 2013 for India based on study estimates and Global Burden of Disease (GBD)[1] estimates for ischemic heart disease (IHD; figures 1A, 1B) and stroke (figures 1C, 1D) among men and women of all ages. To compare to the GBD (which uses an algorithm to re-classify deaths from ill-defined causes), redistributed study estimates were generated from the study by proportionally redistributing deaths classified under ill-defined causes* across other causes of death annually for each 5-year age group. The majority of these ill-defined deaths (78·5%) were >70 years of age. To ensure comparability, both study and GBD estimates excluded deaths < age 15 years and were standardized to the WHO population. For GBD estimates, the shaded area indicates upper and lower 95% confidence intervals. For study estimates, the shaded area indicates upper and lower bounds. The lower bound only includes deaths initially assigned to the same category by both physicians. The upper bound includes deaths initially assigned to a category by either one or both physicians.

*Ill-defined causes: P96, R02, R07, R09, R10, R25, R51–R54, R57–R58, R60–R62, R64, R68, R69, R78, R79, R83, R89, R92–R99

Reference

1 Global Burden of Disease Collaborative Network. Global Burden of Disease Study 2016 (GBD 2016) Results. Seattle, United States: Institute for Health Metrics and Evaluation (IHME), 2017 Available from http://ghdx.healthdata.org/gbd-results-tool (accessed Jan 23, 2018).

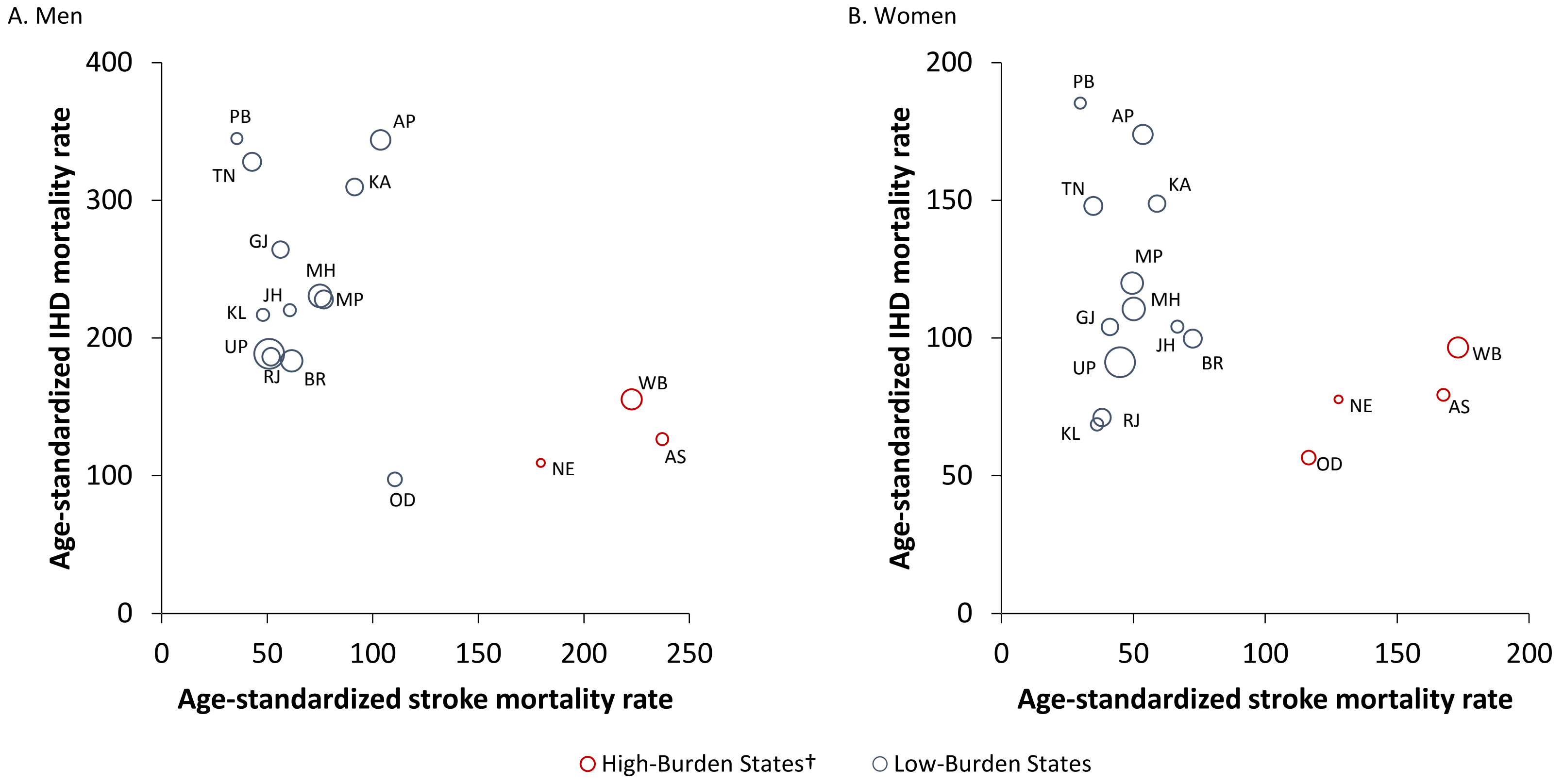

Supplementary Figure S2 Age-standardized mortality rates per 100 000 population for ischemic heart disease (IHD) versus stroke by state for 2010–13 among men and women aged 30–69 years. States and union territories with population < 26 million people are suppressed for clarity. We excluded Himachal Pradesh, Chandigarh, Uttarakhand, Daman & Diu, Dadra & Nagar Haveli, Goa, Lakshadweep, Puducherry, and Adaman and Nicobar Islands due to sparse data. Italicized abbreviations indicate lower income states, which contain about half of India's population.

Abbreviations: AP, Andhra Pradesh; AS, Assam; BR, Bihar; GJ, Gujarat; JH, Jharkhand; KA, Karnataka; KL, Kerala; MH, Maharashtra; MP, Madhya Pradesh; NE, northeast states (Sikkim, Arunachal Pradesh, Nagaland, Manipur, Mizoram, Tripura, Meghalaya); OD, Odisha; PB, Punjab; RJ, Rajasthan, TN, Tamil Nadu; UP, Uttar Pradesh; WB, West Bengal

[†]High-burden states are those with high stroke mortality; all other states are low-burden

High-burden states for male stroke: Assam, West Bengal, Chhattisgarh, and the northeast states (Sikkim, Arunachal Pradesh, Nagaland, Manipur, Mizoram, Tripura, Meghalaya)

High-burden states for female stroke: Assam, West Bengal, Odisha, Chhattisgarh, and the northeast states

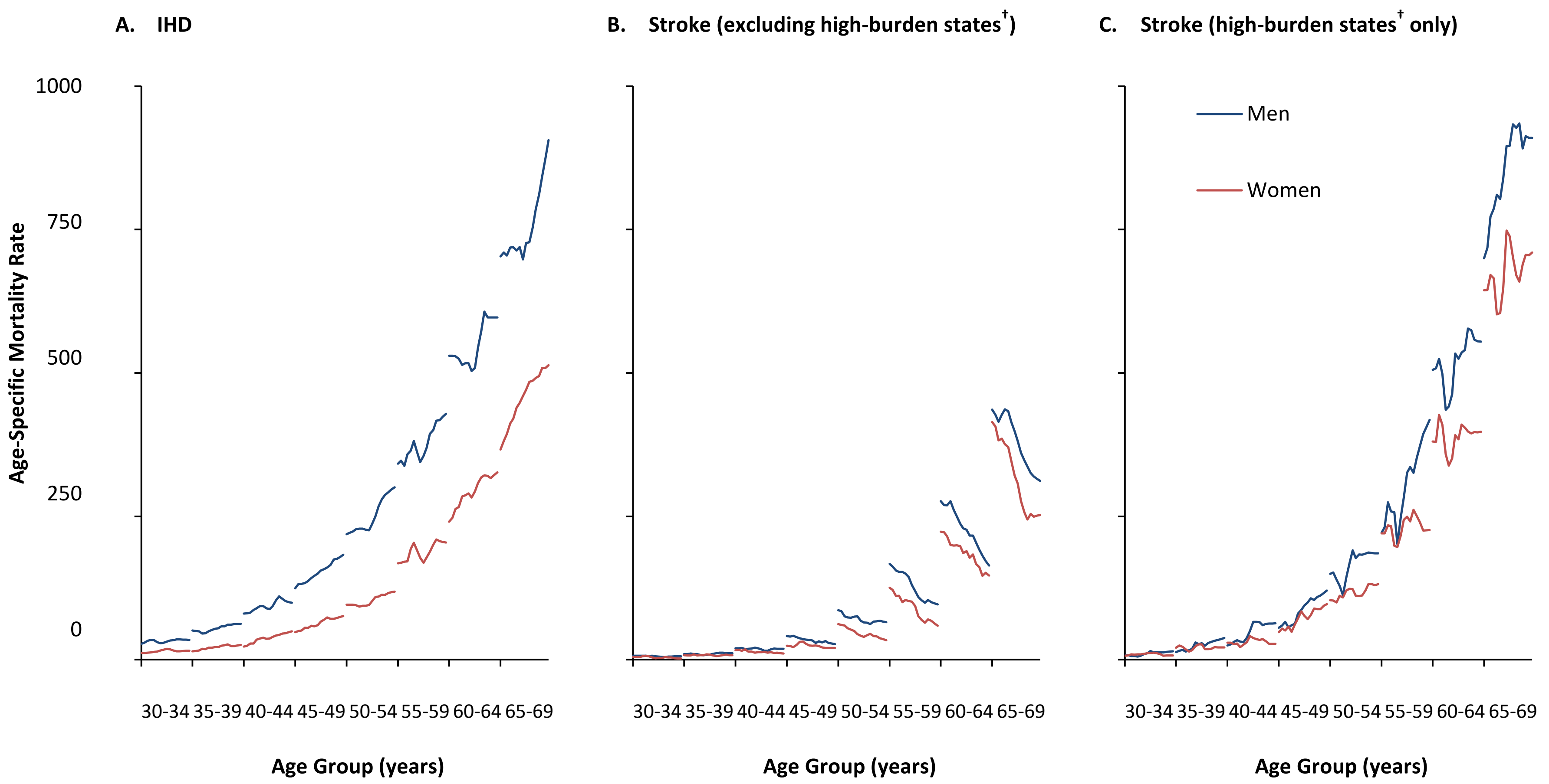

Supplementary Figure S3 Age-specific mortality rates (per 100 000 population) for men and women dying prematurely of ischemic heart disease (IHD; A) and stroke (B, C) by age group. Each line represents the trend from 2000 to 2015.* Stroke mortality rates for high- and low-burden states are shown separately.† All rates are standardized to the World Health Organization population.

*Subnational rates may not average to national rates because subnational and national trends are individually projected to 2000 and to 2014–2015

†High-burden states are those with high stroke mortality; all other states are low-burden
High-burden states for male stroke: Assam, West Bengal, Chhattisgarh, and the northeast states (Sikkim, Arunachal Pradesh, Nagaland, Manipur, Mizoram, Tripura, Meghalaya)
High-burden states for female stroke: Assam, West Bengal, Odisha, Chhattisgarh, and the northeast states

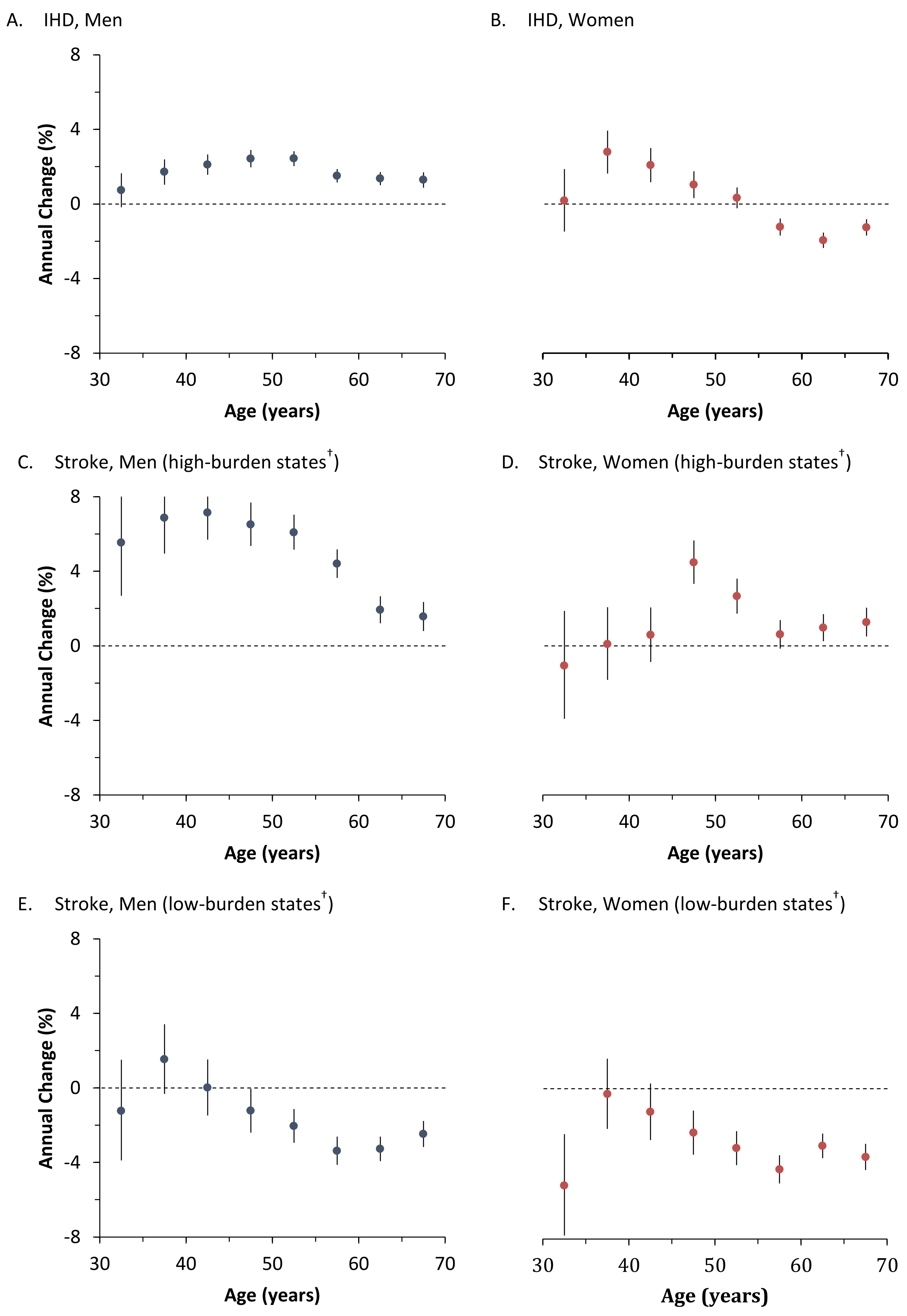

A. IHD, Men
B. IHD, Women
C. Stroke, Men (high-burden states†)
D. Stroke, Women (high-burden states†)
E. Stroke, Men (low-burden states†)
F. Stroke, Women (low-burden states†)
Annual Change (%)
8
4
0
-4
-8
Age (years)
30
40
50
60
70

Supplementary Figure S4 (previous page) Estimated annual changes in age-specific mortality rates by sex for (A) ischemic heart disease (total population), (B) stroke (low-burden states), and (C) stroke (high-burden states only). Error bars represent 95% confidence intervals.

†High-burden states are those with high stroke mortality; all other states are low-burden

High-burden states for male stroke: Assam, West Bengal, Chhattisgarh, and northeast states (Sikkim, Arunachal Pradesh, Nagaland, Manipur, Mizoram, Tripura, Meghalaya)

High-burden states for female stroke: Assam, West Bengal, Odisha, Chhattisgarh, and northeast states (Sikkim, Arunachal Pradesh, Nagaland, Manipur, Mizoram, Tripura, Meghalaya)

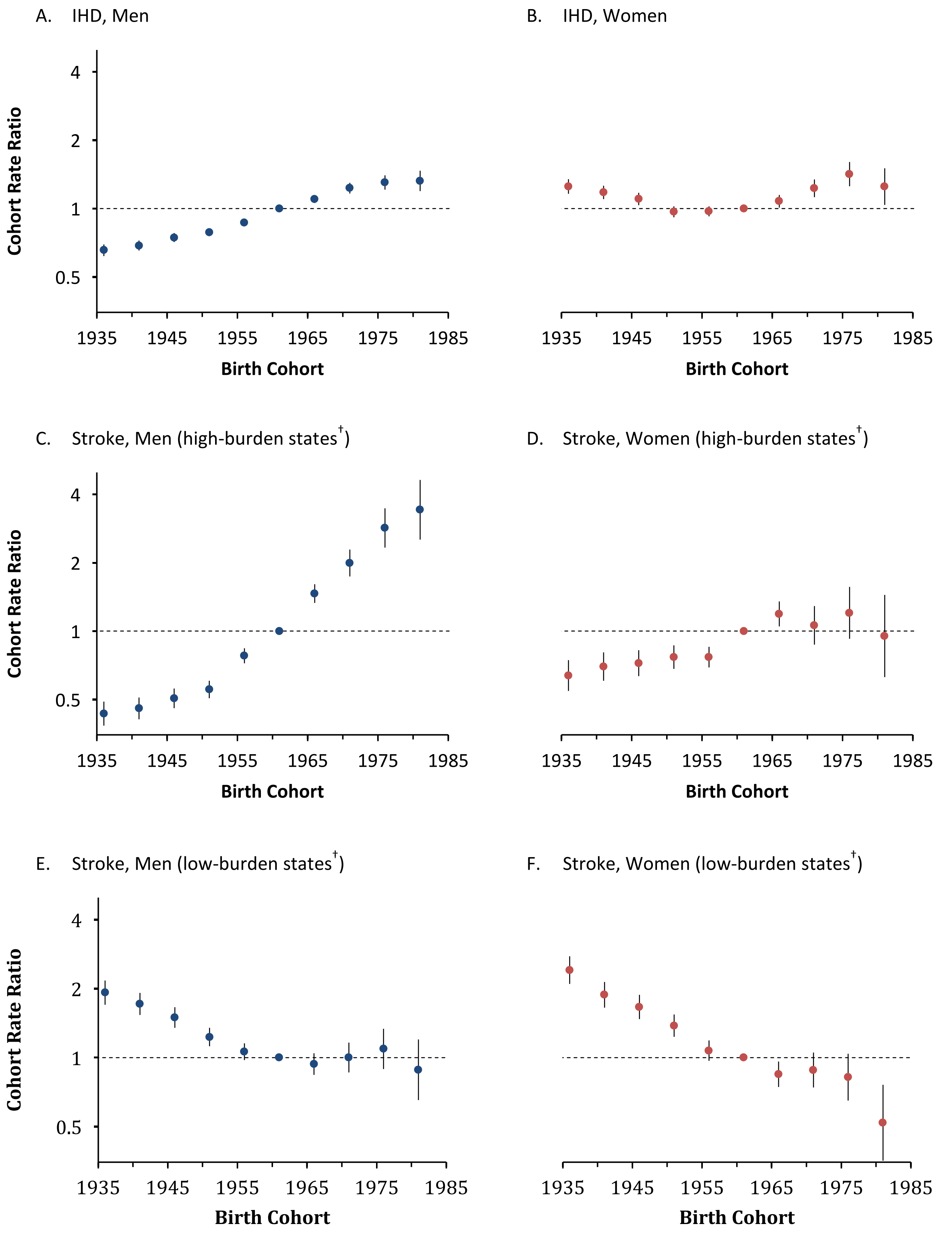

Supplementary Figure S5 Ratio of age-specific mortality rates by birth cohort and sex compared to the 1961 birth cohort for (A) ischemic heart disease (total population), (B) stroke (high-burden states), and (C) stroke (low-burden

states).
Error bars represent 95% confidence intervals. Some error bars are too small to be seen.
†High-burden states are those with high stroke mortality; all other states are low-burden
High-burden states for male stroke: Assam, West Bengal, Chhattisgarh, and northeast states (Sikkim, Arunachal Pradesh, Nagaland, Manipur, Mizoram, Tripura, Meghalaya)
High-burden states for female stroke: Assam, West Bengal, Odisha, Chhattisgarh, and northeast states (Sikkim, Arunachal Pradesh, Nagaland, Manipur, Mizoram, Tripura, Meghalaya)

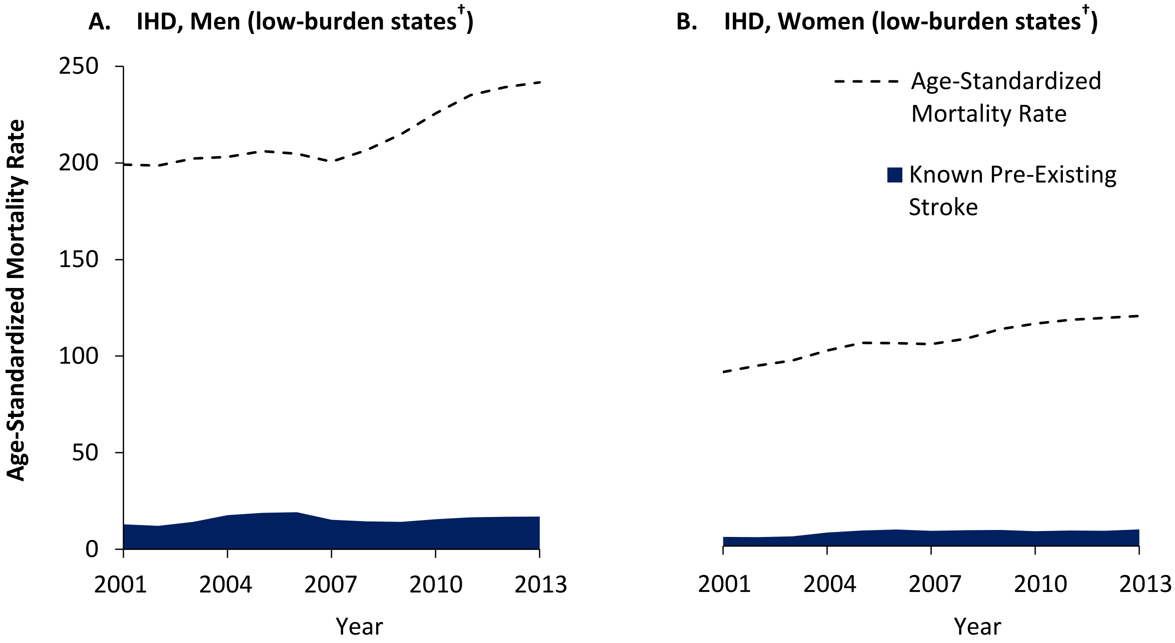

Supplementary Figure S6 Secular trends in the proportion of those with known pre-existing stroke among men and women aged 30 to 69 years dying of ischemic heart disease (IHD) among men (A) and women (B) in low-burden states.† The shaded areas represent proportions overlaid onto age-standardized mortality rates (per 100 000 population). All rates are standardized to the World Health Organization population. The proportion of people with unknown previous stroke status ranged from 4.1–14.2% among men and 3.7–11.6% among women.

†High-burden states are those with high stroke mortality; all other states are low-burden
High-burden states for male stroke: Assam, West Bengal, Chhattisgarh, and the northeast states (Sikkim, Arunachal Pradesh, Nagaland, Manipur, Mizoram, Tripura, Meghalaya)
High-burden states for female stroke: Assam, West Bengal, Odisha, Chhattisgarh, and the northeast states
